# ESF Programme on Functional Genomics 1st European Conference: Functional Genomics and Disease 2003

**DOI:** 10.1002/cfg.332

**Published:** 2003-10

**Authors:** Jo Wixon, Joan Marsh

**Affiliations:** 1 MRC UK HGMP-RC Hinxton Cambridge CB10 1SB UK; 2 John Wiley and Sons Ltd 4th Floor International House Ealing Broadway London UK

## Abstract

In this report from the 1st European Conference of the European Science Foundation Programme on Functional Genomics, we provide coverage of the high-profile plenary talks and a cross-section of the many presentations in the disease analysis symposia and functional genomics technologies workshops.


**Comparative and Functional Genomics**
*Comp Funct Genom* 2003; **4**: 549–557.
Published online in Wiley InterScience (www.interscience.wiley.com). **DOI:** 10.1002/cfg.332

**Feature**

### Meeting Report: ESF Programme on Functional Genomics 1st European Conference: Functional Genomics and Disease 2003

Prague, Czech Republic, 14–17 May 2003

Jo Wixon[1]* and Joan Marsh[2]
[1] *MRC UK HGMP-RC, Hinxton, Cambridge CB10 1SB, UK*
[2] *John Wiley and Sons Ltd, 4th Floor, International House, Ealing Broadway, London, UK*

*Correspondence to:
Jo Wixon, MRC UK HGMP-RC, Hinxton, Cambridge CB10 1SB, UK.
E-mail: jwixon@hgmp.mrc.ac.uk*

### Abstract

**In this report from the 1st European Conference of the European Science Foundation Programme on Functional Genomics, we provide coverage of the high-profile plenary talks and a cross-section of the many presentations in the disease analysis symposia and functional genomics technologies workshops. Copyright © 2003 John Wiley & Sons, Ltd.**

### Plenary presentations

**Leena Peltonen (National Public Health Institute, Finland)** spoke about what can be learned from rare and common disease genes in a genetic isolate. Diseases can be more prevalent in one population, due to a founder effect, when all cases are derived from one original carrier in a small population. The length of an interval of linkage disequilibrium relates to the age of a mutation; in Finnish populations, some regions of LD containing disease genes are 13 cM long; they are relatively young. Using a shared haplotype approach works well in a founder situation. They used it to narrow down the critical region for progressive myeloclonus epilepsy (PME). Using LD allows the use of a relatively sparse genetic map to study monogenic disorders, which is more cost- and time-effective. Her group has mapped ∼20 Finnish rare monogenic diseases using this approach, and in several cases a gene has been cloned. They aim to move on to provide diagnostic tests and carrier screening programmes and have made a chip array of all of these mutations to allow genotyping.

In the case of complex traits, the advantage of isolated populations is that they have a higher degree of genetic homogeneity. There are fewer mutations, and they are easier to identify. There is also a higher degree of environmental homogeneity and lifestyles, diet and culture are far more similar. They have made scans and identified loci in 13 complex diseases, most of which have been verified in other populations. They have found that the idea that once you know where an LD block is you will only need one SNP does not hold true. They do not know how many SNPs per block will be needed, but they often find that it is more than two.

**Hans Lehrach (Max-Planck-Institute for Molecular Genetics, Germany)** used chromosome 21 to illustrate a systems biology approach to investigating disease processes. Most current databases focus on either a single organism or one type of information, e.g. protein structure. The Genome Matrix (**www.genome-matrix.org**) is a new database/interface that will integrate all the information available for a given gene. Inputs include whole-mount *in situ* expression data for all genes on chromosome 21 and an expression map for all these genes in the mouse on post-natal day 2. In due course, the model will include information from knockout studies in model organisms, RNAi experiments and even clinical information, such as

Copyright © 2003 John Wiley & Sons, Ltd.

chest X-rays from a patient with a mutation in the gene being studied.

**Ian Dunham (Wellcome Trust, Sanger Institute, UK)** gave a lovely talk on studying the structural and functional properties of the human genome through the microcosm of chromosome 22. The idea is to combine detailed annotation of both gene and protein expression with analysis of replication timing. DNA is replicated from multiple origins but it is not known whether this is initiated stochastically on open regions of chromatin and then progresses to euchromatin, or whether there is an underlying programme.

**Mathias Uhlen (Royal Institute of Technology, Sweden)** described an ambitious project to create affinity reagents for all known human gene products. Most of the assays are performed on denatured tissue samples, therefore polyclonal antibodies are preferred, but these need to be complemented with monoclonal antibodies to ensure specificity and continued supplies of the reagents. Two of the challenges to be met are the difficulty of making human proteins in *Escherichia coli* and the problem of antibody cross-reactivity. The Swedish scheme is based on protein epitope signature tags (PrEST). A bioinformatic tool searches each full-length ORF for sequences with low homology to the rest of the genome and designs PCR primers automatically, avoiding transmembrane domains. The PCR products are cloned and expressed in *E. coli* and the purified proteins are used to generate antibodies in chickens. The antibodies are used to screen an array of human tissue samples for detailed histopathological analysis on a large scale. This often reveals complex expression patterns, with a protein showing up in a variety of tissues.

The Human Proteome Resource, which was launched in January 2003, plans to isolate the proteins from 14 000 genes in 4 years. Arrays will be prepared from 48 human tissues, the 18 most frequent cancers that occur in Western populations and 60 human cell lines. The aim is to prepare 700 high-resolution images for every protein, showing its relative expression down to the subcellular level in these samples.

**Kelly Frazer (Perlegen, USA)** described the use of high-density oligonucleotide arrays in studying mammalian genomics. They have over 13 billion 25-mer probes, representing the non-repetitive fraction of the human genome, on a series on high-density microarrays.

They have used 248 000 long-range PCRs to amplify single copies of human genome sequences and have identified over 1.7 million SNPs. These have been used to define 175 309 haplotype blocks (where the definition of a haplotype block is one in which 80% of human chromosomes looked at must have one of three patterns).

In comparisons with non-human primates, they found that human and chimp long-range PCR products are generally the same size but that some deletions and insertions occur. A more detailed comparison revealed that the deletions comprise varying amounts of unique and repetitive sequences and that some regions have undergone genomic rearrangement. Using the orang utan as an outgroup, they found insertions and deletions in both the human and chimp genomes and that the majority of rearrangements were randomly distributed.

Comparing a region of human chromosome 21 with six other mammalian genomes, they found sequences conserved in all mammals (common), sequences conserved in three or more mammals (restricted), and sequences conserved only between humans and one other species (uniquely conserved). They believe that the common and restricted sets of genes are actively conserved, whereas the uniquely conserved genes could simply be the result of evolutionary proximity. 16% of the sequence was conserved between human and some mammals, of which 25% was 'uniquely conserved', 50% was conserved in restricted sets and 25% was common. This indicates that $\sim$11% of human genome sequence is actively conserved, which is much more than is generally recognized. To see whether any of the elements in the restricted class were functional, they identified those elements that were present in human and some other mammals and absent in chimpanzee. Transfection analyses showed that these conserved elements do have functional consequences and therefore may be involved in expression differences between species.

Many of the diseases that have the greatest impact on public health are complex disorders that show non-Mendelian inheritance. **Jurg Ott (Rockefeller University, New York)** discussed association analysis for the identification of multiple susceptibility loci in such traits. Linkage analysis emphasizes tracing a disease and a marker being inherited together. For linkage disequilibrium to fall to 50%, one needs SNPs 80 kb apart, which is a very small distance for geneticists, but still

Copyright © 2003 John Wiley & Sons, Ltd. 

means that 20 000 SNPs are required to cover the genome. Haplotype blocks may mean that fewer SNPs may be sufficient; however, genome-wide association studies are still expensive. Association analysis is a more powerful technique than linkage analysis when looking for weak alleles. In case-control association studies, a $2 \times 2$ matrix is plotted of two alleles in cases vs. controls and the $\chi^2$ value indicates the strength of the association. SNPs with the highest value for $\chi^2$ are taken to be the most significant. For a specific tissue, gene expression is compared in cases vs. controls or in normal vs. diseased tissue, e.g. psoriasis. The values of $\chi^2$ are then summed for particular groups, e.g. the five strongest markers. The $p$ values for these sums decrease to a minimum then increase again as the size of the group increases. SNPs that are close to the disease gene increase the value of the association, but SNPs that are further away simply add noise. Therefore, the smallest $p$ value is the statistic of interest. This technique was applied to the analysis of candidate genes in 779 patients with heart disease who underwent angioplasty, comparing those who underwent restenosis with those who did not. The technique provided an answer statistically when normal analysis had failed to get a result. The method can also be used to analyse microarray data and Jurg Ott is starting to extend it to analyse interaction data. Alternative approaches include data mining and combinatorial partitioning.

**Klaus Lindpaintner (Hoffmann-La-Roche, Switzerland)** spoke about the impact of genetics and genomics on drug discovery and development. First, he explained how he sees pharmacogenomics, which concerns many compounds and one genome, and pharmacogenetics, which concerns one compound and many genomes. He pointed out that drugs are a special case of the gene–environment interaction, which may be particularly sensitive to genetic background. There are two distinct entities in pharmacogenetics: the classical, which is related only to drug action, to the absorption, metabolism and elimination of drugs; and the pathology-related, which relates to the underlying disease pathology, a molecular differential diagnosis. These are fundamentally different concepts but both lead to stratification of patients based on a marker.

He is concerned at the lack of systematology and metrics in pharmacogenetics, and proposed some ideas for this, including the assessment of sensitivity and specificity of tests, and the positive and negative predictive values of tests. The TPMT test, for example, can give negative results for some patients who then go on to have side effects.

He feels that pharmacogenetics is likely to enhance the efficacy of drugs, and he does see a role in diagnosis and prognosis, but he feels that it is less likely to contribute significantly to the avoidance of adverse events. The majority of adverse reaction deaths are due to prescription errors, which are not genetically controlled. However, the FDA is happy for drugs to 'do better' and will now permit statements such as 'patients with marker X have a six times higher risk of adverse effects'.

**Jan van Oostrum (Novartis, Switzerland)** explained that the role of the Functional Genomics team at Novartis is to identify the cause of disease and to translate that genomic discovery into drug discovery. The therapeutic areas they are working on include Alzheimer's disease, cancer and chronic pain. They take a multidisciplinary approach, including bioinformatics, model organisms, pathway studies, antisense oligos and RNAi, and use a range of functional genomics technologies, such as arrays and proteomics. They have an in-house cDNA collection of 28 000 predicted full-length cDNAs with very low redundancy, which they have arrayed. They use antisense and RNAi to verify their understanding of the effects of genes of interest, and their proteomics studies use a range of methods, including 2DGE, SELDI and interaction analyses.

**Georg Feger (Serono, Switzerland)** spoke about a high throughput functional analysis of the secretome. Their approach is to identify the 'actors' in the process and demonstrate that they have a pharmacological role. They are interested in the areas of reproductive health, autoimmunity, metabolism, cancer, fibrosis and neurology. The 'secretome', all of the cell-surface and secreted proteins, is a source of therapeutic proteins. They have used a range of bioinformatic approaches to identify all of the human secreted proteins, which represent ~10% of all proteins. The cDNAs of these genes are then transiently expressed in mammalian cells and purified (these can be kept as frozen stocks for ~1 year). They then study these *in vitro* using 50 validated high-throughput cell-based assays (with varying levels of complexity). This approach identified 100 potentials, which were narrowed down to 10 valid candidates (since the therapeutics are

Copyright © 2003 John Wiley & Sons, Ltd. 

endocrine, they need to be active *in vivo*). From their assay set, they look for genes showing similar patterns of results to infer related function for genes of unknown function, and then confirm these inferences using more detailed analyses.

### Disease analysis symposia

### Infection and host–parasite interaction

**Peter Jungblut (Max-Planck-Institute of Infection Biology, Germany)** spoke about his group's efforts to elucidate the proteomes of microorganisms. In proteomics, an important goal is to increase the number of detectable proteins; he outlined various technical refinements that his group and others have made, noting that the use of complementary technologies, such as 2D gels and ICAT can be very useful. In their hands, these two techniques find only a small proportion of proteins in common, but do show matching quantitation, in those cases where all of the spots on the 2D gel that come from the protein of interest can be accounted for. They have detected ∼20% of the proteins of *Helicobacter pylori*, whereas in *Mycobacterium tuberculosis*, they have only identified ∼10% of the 350 proteins; they hope to raise this to 30% using the ICAT approach. They have also made comparisons between non-virulent and virulent mycobacteria, comparing *M. bovis* BCG Chicago and *M. tuberculosis* H37Rv revealed six new ORFs and 32 spots unique to the virulent strains. The data from all of their studies, which cover 10 microorganisms and some eukaryote tissues, are available in a relational database from: **http://www.mpiib-berlin.mpg.de/2D-PAGE**.

**Rino Rappuoli (Chiron, Italy)** presented a genome-based approach to new vaccines. They are working to develop vaccines against meningococcus; this bacterium causes 350 000 cases/year, 35 000 of which are fatal. Although there is a long chain polysaccharide vaccine for meningococcus C, this is not possible for the B type, as its capsular polysaccharide is identical to a human one, so it is recognized as 'self'. A membrane protein, PorA, has been used as a target, but due to the variability of this protein, the vaccine is strain-specific. His team have collaborated with TIGR and Oxford University to sequence the genome of *Neisseria meningitidis*, with a view to aiding vaccine design. This resulted in 600 potential vaccine candidates, 350 of which they have successfully expressed in *E. coli*. Purification of the proteins, followed by immunization of mice and sera testing, successfully identified 91 novel surface-exposed proteins, of which 28 showed bactericidal activity. To avoid producing a strain-specific vaccine, they looked for those proteins that were conserved across strains; this reduced the list to five vaccine candidates, which have now gone into trials. He feels that this approach has been very productive and plans to apply it to other pathogens.

**Neil Hall (Wellcome Trust Sanger Institute, UK)** described the insights into antigenic variation in malaria that have been afforded by the recently completed genome sequences of *Plasmodium falciparum* and *P. yoelii* and the other related genomes that are in progress.

**Christoph Dehio (Basel, Switzerland)** described studies of the expression of *Bartonella henselae* genes, and human genes, during infection, done in collaboration with Siv Andersson's group, who sequenced the genome of this bacterium. Bacterial genes induced during infection include transport and secretion factors, including the type IV secretion system, which mediates most of the effects that the *vir* genes have on endothelial cells. Looking at the expression of human genes in the endothelial cells infected with wild-type or *vir* mutant bacteria showed that about 10% of the genes on the array were affected after 6 h (early infection) and just over twice as many were affected at 30 h (well-established infection). Some genes were more strongly induced by the *vir* mutant infection, implying that they could be repressed by the *vir* pathway, whereas others were more strongly induced by the wild-type infection, which could indicate that they need the *vir* pathway for the best effect to be obtained.

### Oncology

**Julio Celis (Copenhagen, Denmark)** spoke about the application of proteomics to cancer research. The inherent challenge in this is detecting the ∼300 000 proteins that are encoded by the human genome (allowing for all the splice variation and post-translational modifications). His group is part of the Danish Initiative for Breast Cancer Proteomics. Breast cancer is the most common cause of cancer-related death in women worldwide. The

Copyright © 2003 John Wiley & Sons, Ltd.

disease is very heterogeneous, so there is a need to profile patients to allow for the provision of corresponding treatment. They aim to find markers for classifying types of histopathology, and patient stratification, for early detection and for monitoring progress and response to treatment. They obtain samples of normal and diseased breast tissue and lymph node metastases rapidly after mastectomy and make frozen stocks. Fresh tissue profiling (including proteomics, transcriptomics and pathological assays) is done using portions of the same sample. The normal tissues are used to build up a profile of normal background, and connective tissue samples have been profiled so that this contaminating signal can be subtracted from breast tissue data. They only resort to laser capture microdissection if they are desperate. They make extensive use of antibodies to verify the differences seen on the gels using tissue slides.

**Olli-Pekka Kallioniemi (University of Turku, Finland)** discussed the use of microarrays in cancer studies. He noted that while microarrays have successfully been used to classify cancers without knowledge of the underlying biology, which is good for diagnosis and prognosis, they have only given a few clues towards cancer mechanisms and therapies. He argued that no single array platform can provide the answer to the bottleneck in converting genomics data into new targets for therapy. He described three key array-based approaches that his group have applied to cancer research. In the first, traditional expression arrays were combined with comparative genome hybridization to identify 270 genes whose expression was significantly influenced by copy number in breast cancer; all of the known important breast cancer fell in this group. They have also used cell-based arrays (arrayed cells, or arrayed biomolecules overlaid with cells) to look at the effects of overexpression, or suppression of expression, of selected genes. The third approach is to use tissue arrays (with up to 1000 tissue samples on an array) to explore prognostic implications. These have been hybridized with antibodies to discover which cancers are expressing genes of interest.

### Hereditary disease

**Han Brunner (University Medical Centre Nijmegen, The Netherlands)** described how many complex syndromes share overlapping features, but also have distinctive features, so the question is whether they are the same condition or not? The clinicians who first described these syndromes were either 'lumpers' or 'splitters' and debated this question vigorously. Modern genetics is revealing that diverse syndromes may be due to mutations in the same gene. Differences arise from mutations in different parts of these genes, affecting protein function or tissue-specific expression.

The converse situation is when one disease may be associated with many genes. Fanconi's anaemia results from mutations in genes encoding members of the FA complex, all of which are involved in DNA replication. This is a common phenomenon: he estimates that half of genetic conditions may involve multiple genes for a single disorder because most proteins are involved in interactions. Phenotypes define functional modules, not genes. Walker–Warburg syndrome comprises muscular dystrophy, brain malformations and eye abnormalities; it is the most severe of a spectrum of diseases. The proteins affected by the mutations underlying these diseases do not interact directly but they all act in a single pathway governing *O*-mannosylation.

**Andrea Ballabio (Telethon Institute of Genetics and Medicine, Italy)** described deficiencies in the activities of specific sulphatases that result in several types of human disease. Ichthyosis is an X-linked recessive disorder that manifests as scaly skin. The steroid sulphatase gene responsible was cloned in 1987; 85% of patients have deletions in this gene, some of which span other genes, resulting in up to six disorders. One is chondrodysplasia punctata, which is due to mutation of another sulphatase gene. There are 14 known human sulphatases; four of which cluster on the distal arm of the X chromosome. They have conserved sequences, especially a cysteine in the catalytic site.

Multiple sulphatase deficiency (MSD) is a condition in which the activity of all sulphatases is seriously reduced. It is caused by a single, autosomal recessive gene defect and has a severe phenotype. The mutation was found in the sulphatase modifying factor 1 gene. Mutations in this gene were found in every patient tested, all involving a splice site, creating a missense transcript. Sulphatases need post-translational modification to change the catalytic site cysteine into

Copyright © 2003 John Wiley & Sons, Ltd. 

formylglycine. Vertebrates have two genes for sulphatase modifying factors but the mutations found so far are restricted to SUMF1. Recent work has shown that the sulphatases within a cell compete for the SUMF activity. This observation is having a major clinical impact. Previously, patients were treated with enzyme replacement therapy, but 80% of sulphatases produced in cell lines are inactive; addition of SUMF1 restores sulphatase activity.

**Daniela Toniolo (San Raffaele Scientific Institute, Italy)** described animal models for non-specific mental retardation. About 2% of the human population is mentally retarded, with an IQ below 70, and about half of these cases are due to genetic causes. It may occur as part of syndrome, but in many patients, mental retardation is the only clinical manifestation. There is an excess of male patients; 14 genes linked to mental retardation have been located on the X chromosome, one of which is the Gdi1 gene.

GDI1 is a protein involved in the Rab-GDP/GTP cycle; it binds to inactive Rab-GDP and maintains it in a soluble, inactive form. In the brain, $\alpha$GDI interacts with Rab3 proteins in synaptic vesicle fusion and neurotransmitter release. She generated a mouse in which the Gdi1 gene was knocked out. The mice were viable and fertile and appeared normal in many behavioural tests. The level of aggression in male mice was reduced, but the Gdi knockouts showed normal olfactory abilities and testosterone levels. The Gdi knockout mice performed normally in a spatial memory test but failed in a working memory test. After further tests, she concluded that the mice were unable to associate events that were separated by a short time, due to specific cognitive deficits in working and associative memory. Histological analysis showed that the Gdi knockout mice had a reduced number of synaptic vesicles. There were sufficient for the mice to respond to a single stimulus, but the mice needed a prolonged inter-test interval to replenish synaptic vesicles before they could respond to the next test.

### Functional Genomics Technologies Workshops

### Proteome and protein–protein interactions

This session was opened by **Ivan Lefkovits (University of Basel, Switzerland)** who is studying the proteome of lymphocytes. This is a huge challenge because an active lymphocyte contains about 40 000 mRNA molecules and about 1 000 000 000 polypeptide chains. A single mRNA can give rise to 44 polypeptide spots on a 2D gel and there may be 5000 different peptides present in a cell at a given time. He plans to focus on the rare proteins that may function under specific (physiologically unfavourable) conditions. More details on this work can be found in his review (in this issue, p. 531–536).

**Michael Dunn (Institute of Psychiatry, UK)** described proteomics in another immunological setting, heart transplantation. At present, there is no non-invasive test to predict which transplant patients will reject the donated hearts, either acutely or chronically. His team are looking for disease markers directly in plasma using a variety of techniques, e.g. 2-DGE, LC–MS, protein or antibody arrays and a SELDI–MS chip-based assay. A major problem is the dynamic range, which is 1012 for the plasma proteome: 99% of the protein mass of plasma comprises only 22 proteins and all the interesting markers are lost in the other 1%. One approach is to use antibody depletion, then look at the remaining proteins. His approach is called REMAP. Proteins from sequential endomyocardial biopsies are analysed by 2-DGE to detect alterations in expression. These markers are then examined to see whether they are diagnostic or predictive for acute rejection. Candidate proteins are identified and antibodies are raised against them. The antibodies are used to detect whether the rejection markers are present in plasma and, if so, whether they can be used to monitor acute rejection. This approach has produced two candidates, $\alpha$-tropomyosin and $\alpha$-crystallin B chain, both of which show a doubling of concentration in the plasma of patients experiencing acute rejection.

A similar approach has shown that vimentin, an intermediate filament protein from endothelial cells, acts as a marker for patients at risk of developing chronic rejection. The anti-vimentin antibody titre is correlated with survival.

Switching the emphasis to cancer, **Pranav Sinha (Universitatsklinikum Charite, Germany)** explained that there are 110 000 drug-resistant tumours/year in Germany. These arise through the overexpression of P glycoprotein (which transports the drug out of the cell), or other enzymes that conjugate the drug so that it is excreted, or that

Copyright © 2003 John Wiley & Sons, Ltd. 

sequester it within the cell so that it cannot reach its target. He cultured cell lines in the presence of anticancer drugs to induce resistance, then looked for altered patterns of protein expression compared to the parent cell line. Increased concentrations of several classes of proteins were observed, including chaperones, creatine kinase and annexin. He also saw that gastric and pancreatic cancers showed different protein profiles.

### Transcriptome

**Wilhelm Ansorge (EMBL, Germany)** spoke about the production of a human genome chip. His team, in collaboration with colleagues at RZPD, selected the RZPD3 Human Unigene Set and arrayed these onto a chip; 48 000 (~90%) of them have been sequence verified so far. His team took great care in comparing alternatives for each part of the process, from surface chemistry, to pin loading onto the chips, to labelling techniques. They use standardized experimental conditions and adhere to the MIAME guidelines, and the bioinformatic support for the project has been developed in collaboration with the EBI.

In addition to this whole-genome chip, they have other projects, such as the stem cell division microarray. This array of candidate genes is screened with RNA from highly purified human HSC populations. This is a very challenging task; many cells are needed to yield enough RNA for each experiment. Using a pilot chip, they have optimized their approach, particularly the RNA isolation method, and can now use just 100 ng RNA per array.

Another goal is to produce a human protein chip, an antibody array for protein profiling, which would allow the detection of post-translational modifications. To produce the antibodies, they inoculate mice with 8–10 antigens and fuse the collected B cells with myeloma cells. They obtain single cell cultures and use an ELISA approach to verify the antibodies before use on the array.

**Franco Pagani (International Centre for Genetic Engineering and Biotechnology, Italy)** described two new types of disease-causing mechanism that perturb RNA processing. The first occurs in ataxia-telangiectasia and involves a defect in an intronic splicing processing element. The second is an exon-skipping defect observed in cystic fibrosis. He suggests that splicing regulatory elements occur throughout introns and allow the RNA polymerase to jump along, rather than reading processively. Thus, many polymorphisms deep inside introns that were thought to be benign may affect RNA splicing efficiency.

**Frank Holstege (University Medical Centre Utrecht, The Netherlands)** gave an excellent discussion of methods for maximizing genome-scale data. He identified four challenges: (a) the rate of generation of hypotheses is faster than the rate of verification; (b) the problem of data quality, as high-throughput techniques are associated with high error rates; (c) functional annotation; and (d) genome annotation. These can be addressed by integrating information from publicly available datasets, such as from microarrays, protein databases and phenotype descriptions.

High-throughput protein interaction data include many false positives that need to be eliminated. One method is to compare the interaction data with expression data; if the mRNAs are co-expressed spatially and temporally, then it is more likely that the proteins interact. Using a cosine function to represent the degree of co-expression, he was able to discount half of the proposed protein–protein interactions in a dataset as false positives.

Protein interactions can be used to characterize unknown proteins. For pairs of proteins, information on the first was used to predict the function of the second, which was then tested. By this approach, he was able to assign a function to 326 uncharacterized yeast ORFs.

### Advances in microarrays

**Anne-Christine Syvänen (Uppsala University, Sweden)** described how her group use microarrays to genotype SNPs. They use a DNA polymerase-assisted primer extension 'minisequencing' approach, with primers covalently attached to the array as a 14 × 14 array of subarrays of 50 spots. Up to 80 samples can be analysed for 200 primer sets per slide, generating 16 000 genotypes per slide. The minisequencing approach is sensitive enough to be used for quantitative SNP detection, and they have been able to detect alleles with frequencies below 1% in a pooled sample. They have recently developed a four-colour tag microarray system for multiplex genotyping and quantification of SNPs by primer extension,

Copyright © 2003 John Wiley & Sons, Ltd. 

in which the tagged products are captured on an array of complementary tag-oligos.

**Ulf Landegren (Rudbeck Laboratory, Sweden)** spoke about the padlock and proximity probe systems that have been developed by his group for *in situ* and array-based analyses.

Padlock probes can be used to interrogate thousands of SNPs in solution. The products can be specifically amplified, and then identified using a tag microarray. This method avoids the cross-reaction problems of multiplexing using PCR, and has been selected for use by the US Haplotype Map project. The proximity ligation system is designed for measuring protein expression, by generating specific reporter DNA sequences that can be amplified and detected. For more details on this work, see his review in this issue (p. 525–530).

**Ivo Gut (Centre National de Genotypage, France)** discussed the tools available for genomic epidemiology. His group have a DNA collection with 100 000 samples representing a range of pathologies. For small-scale studies, they look for disease-causing SNPs by resequencing candidate genes. Once a polymorphism is found and validated, they switch to high-throughput analysis. For medium-scale studies, they use TaqMan or Amplifluor for SNP genotyping, and for high throughput they use the GOOD assay with MALDI–MS detection. Each system has its advantages and disadvantages; they select which one to use based on those, and use other platforms for particular purposes.

They have found that some alleles are population-specific, so knowledge of 'background' is important. They often see more than the three common haplotypes frequently mentioned in other studies, and he thinks that more SNPs will be needed for genome-wide studies than is often quoted. They are also looking at detecting methylation of DNA, but this needs good calibration curves, as there is a degree of methylation at any one site, unlike SNPs, which are absolute.

### Model systems and knockout

**Christophe Echeverri (Cenix Bioscience, Germany)** discussed the use of RNAi screening to identify a new medicine. His team used RNAi screening to identify new cell division genes. Their automated system designed long dsRNA molecules to target *C. elegans* genes, using data from ACeDB, which it updated each time the annotation was updated. The dsRNAs were microinjected into worms and F1 early and late embryos, and larval and adult F1 progeny were monitored using a high-magnification time-lapse digital microscope. Their screen covered 99.2% of the predicted genes and identified 47 defects. Knockdown of 9% of the genes resulted in a phenotype, half of which where early embryonic, with the others being distributed across the other three stages.

They have carried out a range of assays in *Drosophila*, including of mitotic index and signal transduction. In their latest screen, which took 2 weeks, they have found 19 new components of the cellular anti-viral and RNAi response.

They have also been working with short dsRNAs and he claims that with their modified design criteria, Cenix have raised the success rate of this approach from 60% to around 90%.

**Thomas Rudel (Max Planck Institute for Molecular Genetics, Germany)** spoke about high-throughput RNA interference in mammalian cells. About 30–40% of siRNAs are ineffective in mammalian cells and any effects need to be validated, which makes the approach cost-intensive. They validate their knockdown using real-time quantitative PCR. Typically, they either see ∼80% knockdown or it does not work. The next challenge is to observe the phenotype, there are many assays to be done, and some phenotypes only show up in detailed 'zoomed-in' tests, hence there is a need for automated assays and microscopy.

His group is part of a network, the European Union for RNA Interference Technology (EURIT: **http://www.eurit-network.org**), which aims to do global RNAi screening in mammalian cells. They have four initial projects, to make a collection of published siRNAs, to make a library for vector mediated RNAi (psiRNA), to generate an siRNA design tool and a platform for collection and exchange of siRNAs.

### Bioinformatics

**Alfonso Valencia (Centro Nacional de Biotecnologia, Spain)** spoke about ways to reconstruct protein interaction networks. Across a range of genomes, for ∼40% of genes, we have no clue as to their function from sequence homology, so we need to use other information. One way to do this is to use information on protein–protein interactions. He

Copyright © 2003 John Wiley & Sons, Ltd. 

described the SUISEKI information extraction tool, written by his group, which they applied to PubMed abstracts to obtain protein names and information on their protein–protein interactions. This is being used in collaboration with the EBI to generate a new interaction database called INTACT. He also described other approaches to inferring gene function, including gene neighbourhoods, gene fusions and co-evolutionary approaches. By verifying predicted interactions using the literature, he has found that combining these methods increases the confidence of predictions, and that interactions predicted independently by three methods are much more likely to be true.

**Pierre-Alain Binz (Swiss Institute of Bioinformatics, Switzerland)** discussed issues for bioinformatics in the field of proteomics. He pointed out that when starting a proteomics project it is important to think about the questions that you want to answer before choosing the approach you use. The technologies available (classical 2DGE, MudPIT, ICAT and SELDI) produce quite different results. Bioinformatics can contribute at all stages of the process, from laboratory information management systems (LIMS), to signal detection, to data interpretation, to databases. One such tool provided by the team at SIB is 'Melanie', an image analysis tool for 2D gels, which identifies proteins that vary. He urged people to think about 'what is correct?' There are many mass spectrometry systems and many search tools, with variable parameters, so it is quite possible to get different answers from the same data. He recommends thinking about the confidence that can be placed in the results, e.g. by checking to see whether the proteins that have been identified should be expressed in the organelle or tissue under study.

**Peer Bork (EMBL, Germany)** spoke about the application of bioinformatics to molecular medicine. His group have taken a range of approaches to this, several of which relate to improving the annotation of protein function. They use their SMART tool for domain discovery of genes, which can suggest a function in disease. Their STRING tool performs context analysis of genes, including the phylogenetic co-occurrence, genomic neighbours and gene fusion approaches. They have used their tools for literature and data mining (MESHC and MESHD) with MESH terms for phenotype, and chemistry and GO terms. They try to extract molecular information and link terms to terms. This system was applied to 100 known disease genes, having taken out those papers that linked the genes to diseases, and was able to link many of the genes with the right diseases. G2D is a database of candidate genes that they have mapped to diseases in this way (Candidate Genes to Inherited Diseases: **http://dove.embl-heidelberg.de/g2d/**).

They have also looked at coding repeats in cancer, investigating a link between coding microsatellites and colorectal cancers, in which mismatch repair mutations are common. The repeats are often incorrectly replicated, becoming longer or shorter and resulting in a frameshift in the gene. They made a genome-wide search for coding microsatellites and then looked for those that were expressed in cancer cells. They then looked for cancer cell-specific products caused by the shifts, as these might be displayed on the cell surface, because they are foreign, and so could be an ideal target for antibodies against the cancers. To date, they have ~30 candidates.

Copyright © 2003 John Wiley & Sons, Ltd.

